# The potential of Logistics 4.0 technologies: a case study through business intelligence framing by applying the Delphi method

**DOI:** 10.3389/frai.2024.1469958

**Published:** 2024-10-17

**Authors:** Joaquim Jorge Vicente, Lurdes Neves, Inês Bernardo

**Affiliations:** ^1^CIGEST – Centro de Investigação em Gestão, Business and Economics School, Lisbon, Portugal; ^2^CEGIST – Centro de Estudos de Gestão, Instituto Superior Técnico, Universidade de Lisboa, Lisbon, Portugal

**Keywords:** Logistics 4.0, business intelligence, Delphi method, decision-making, case study

## Abstract

**Introduction:**

The growing competitiveness and the importance of data availability for organizations have created a demand for intelligent information systems capable of analyzing data to support strategy and decision-making. Organizations are generating more and more data due to new technologies associated with Industry 4.0 and Logistics 4.0, making it essential to transform this data into relevant information to streamline decision-making processes. This paper examines the influence of these technologies on gaining a competitive advantage, specifically in a logistics company, which is scarce in the literature.

**Methods:**

A case study was conducted in a Portuguese company using the Delphi method with 61 participants—employees who use the company’s integrated BI tool daily. The participants were presented with a questionnaire via the online platform Welphi, requiring qualitative responses to various statements based on the literature review and the results of semi-structured meetings with the company.

**Results:**

The study aimed to identify areas where employees believe more investment/ development is needed to optimize processes and improve the use of the BI tool in the future. The results indicate that BI is a crucial technology when aligned with a company’s objectives and needs, highlighting the necessity of top management’s involvement in optimizing the BI tool. Encouraging employees to use the BI tool emerged as a significant factor, underscoring the importance of leadership in innovative projects to achieve greater competitive advantage for the company.

**Discussion:**

This study aims to understand the importance of Business Intelligence (BI) and how its functionalities should be adapted according to a company’s strategy and objectives to optimize decision-making processes. Thereby, the discussion focused on the essential role of BI technologies in leveraging the company’s competitive advantage.

## 1 Introduction

### 1.1 Background of study

The business landscape is increasingly shifting toward digitalization, focusing on overcoming challenges and complexities while improving accessibility. This transformation integrates automated systems and technology to enhance operational processes and efficiency. Digital technologies enable businesses to manage data more effectively, streamline operations, and reduce human errors, resulting in faster task completion (Acioli et al., 2021).

Industry 4.0 (I4.0) technologies are pivotal in the logistics sector, driving a shift from manual processes to automation and digitalization and they enhance performance, operational efficiency, and the development of innovative business models, products, and services (Atumonye, 2022). In logistics, advanced technologies facilitate the creation of automated systems and remote access controls, improving efficiency in logistics operations (Schumacher et al., 2016). As technology rapidly evolves, companies strive to keep pace, recognizing the need for technological systems that align with organizational planning and strategies. This trend has led to a surge in information systems projects aimed at aligning Information Technology (IT) with company strategies.

These information systems projects, particularly in Business Intelligence (BI), are instrumental in improving decision-making quality and generating value. However, they require substantial investments in systems and resources. As investment levels rise, it becomes essential for organizations to understand the potential benefits and unique value that these systems can provide.

The economic, political, social, environmental, and technological transformations that have emerged and delineated the main characteristics of contemporary society have inevitably induced shifts in the paradigms of organizational management and work processes. Given the complexity and ambiguity of operational contexts, the strategic nature of decisions and responses has become an integral part of the daily reality of organizations, including the role of leaders. Thus, considering the systemic and contingent dimensions that define and characterize each organization, particularly educational institutions, the exercise of leadership and the requisite skills have undergone continuous redefinitions. This evolution ensures an active and decisive contribution to the ongoing enhancement of the effectiveness and efficiency of the organizational system as a whole.

In this context, transformational leadership has been highlighted and portrayed in the literature as a differentiating factor for success within organizations, showing positive correlations with various performance levels—individual, group, and organizational (Judge and Piccolo, 2004; Wang et al., 2011).

Additionally, associated with the numerous changes and upheavals in the environment, there are ethical and moral dimensions that influence the exercise of leadership. Therefore, leadership must be grounded in both the establishment of a vigorous and empowering emotional connection between the leader and followers and in moral and ethical principles that are socially and institutionally accepted and expressed through the existing organizational culture. This includes the values, beliefs, and ideals cultivated and expanded within organizations.

Given the importance of ethics and morals in organizations and the crucial factors underlying their development, Maier (2002) noted that leadership is among the most significant contributors to this objective. The author also emphasized the responsibility of leaders in charting the course forward and in fostering strategies that build trust, encouragement, and reinforcement of excellent performance within the organizational climate. This approach aims to ensure that organizations managed by these leaders become more humane, respectful, responsible, and ethical, ultimately serving as true examples of altruism—prioritizing the well-being of others. It is also important to see this in BI.

### 1.2 Motivation

In today’s competitive business environment, data and information are crucial for guiding decision-making processes and enhancing corporate performance. Companies are compelled to improve their operations to maintain a competitive edge, using data to generate insights, manage risks, devise strategies, cut costs, and streamline processes (Sonda, 2024). Executives acknowledge the importance of the ongoing “information revolution” and are increasingly investing in IT, seeking assurances that these investments will provide returns (Rodrigues, 2017). IT has evolved from a supportive role to a critical source of knowledge and competitive advantage, impacting both operational and strategic levels.

BI systems exemplify this shift by offering powerful tools for data analysis and managerial decision-making. These systems scrutinize databases to help managers develop effective strategies through data processing and analysis (Olaoye and Potter, 2024). BI systems organize data into actionable insights, providing a comprehensive view of business performance through visualization techniques and detailed analysis (Angeloni and Reis, 2006). Industry analysis, such as Agiu et al. (2014), anticipate continued growth in BI system adoption due to their evident benefits in improving decision-making and operational efficiency.

The latest projections from Gartner, Inc. (Table 1) reveal that global spending on IT is expected to reach $5 trillion in 2024, representing a 6.8% increase from the previous year. 2024 is anticipated to be a pivotal year for the sector, with IT services expected to become the largest segment of IT spending for the first time. Total spending on IT services is forecasted to increase by 8.7%, reaching $1.5 trillion, driven by investments in projects aimed at enhancing organizational efficiency and optimization.

**Table 1:** Worldwide IT spending forecast (millions of USD).

	2023 spending ($)	2023 growth (%)	2024 spending ($)	2024 growth (%)
Data center systems	243,063	7.1	261,332	7.5
Devices	699,791	−8.7	732,287	4.6
Software	913,334	12.4	1,029,421	12.7
IT services	1,381,832	5.8	1,501,365	8.7
Communications services	1,440,827	1.5	1,473,314	2.3
Overall IT	4,678,847	3.3	4,997,719	6.8

Source: Gartner (2024).

Despite substantial investments, project success is not guaranteed. It depends not just on the systems or technology but also on factors like implementation, adoption, and effective utilization. According to Standish Group Report (2014), 31.1% of information systems projects are terminated before completion, and nearly half of ongoing projects exceed their budgets, with final costs averaging twice the initial estimates.

The main motivation to develop this work lies on the impact that BI systems influence the competitive advantage of a logistics company, which is scarce in literature.

### 1.3 Aim and problem formulation

In logistics and supply chain operations, companies are rapidly adopting essential I4.0 technologies, such as Big Data, Cloud Computing, and BI. These technologies are being utilized across various areas, including planning and scheduling, supply chain management, production, and delivery and returns. The adoption of these technologies is expected to improve logistics processes by enhancing efficiency, reducing lead times, and optimizing workflows (Roshid et al., 2024). Leveraging the interconnected nature of I4.0 allows for the implementation of innovative systems that use real-time data to intelligently support decision-making processes.

The use of data within enterprises is becoming increasingly crucial in the modern world. However, a significant amount of this data goes unused and therefore loses value. BI plays a vital role in the fourth industrial revolution (I4.0) by utilizing data and information to facilitate informed decision-making, and it will continue to do so in the future according to Faber’s research in Faber (2024).

### 1.4 Research question

This study examines the influence of these technologies on gaining a competitive advantage, specifically within a prominent Portuguese logistics company. The central research question guiding this study is: What impacts does BI have on competitive advantage in a logistics company?

This question explores the potential benefits of BI technologies for organizations, specifically in terms of enhancing strategic positioning, improving decision-making processes, increasing operational efficiency, and fostering innovation.

To fully address this topic, the study has set three main objectives. Firstly, to determine whether the logistics company being studied is aligned with its goals and objectives. Secondly, to understand the extend which the BI technologies integrate with overall strategy and contribute to competitive positioning, and to gain an understanding of current usage and challenges faced in order to enhance future utilization. The third objective is to identify key areas for improvement and development within the BI tool. This will optimize processes and improve decision-making, ultimately giving the company a competitive edge through BI technologies.

The data gathered from participants’ responses will provide valuable insights and actionable recommendations for the company to strategically position itself in the market using effective BI technology.

### 1.5 Document structure

The document is structured into several sections. This structure ensures a comprehensive exploration of the research topic. Literature Review is described in section 2. This section reviews existing literature in the key topics in study. Methodology can be found in section 3. This section explains the research method used in the study and describes how data was collected. Section 4 presents the Case Study following by section 5 with a Discussion of the results. Finally, in section 6, conclusions are reported and summarizes the findings and limitations of the study and suggests areas for future research.

## 2 Literature review

### 2.1 Industry 4.0

In 2011, the term “Industry 4.0” was introduced by a team from various backgrounds. This term refers to the digital revolution and has gained significant attention from companies in different industries (Tubis and Grzybowska, 2022).

The fourth industrial revolution, known as “Industry 4.0,” is marked by the integration of advanced technologies such as the Internet of Things (IoT), real-time data management and analysis, and automated machinery into industrial systems (Hasnan and Yusoff, 2018). According to Bag et al. (2021), I4.0 technology emphasizes digitalization to enhance company operations through the deployment of emerging technologies and automated systems. In the contemporary digital landscape, logistics companies leverage online services via web servers and applications, utilizing these digital technologies to effectively manage and access data (Cichosz et al., 2020).

Several countries, including the United States, China, and Japan, have adopted strategies to bolster their manufacturing sectors through technological advancements. Portugal is also advancing in this area with its I4.0 plan, which comprises 60 initiatives from both public and private sectors aimed at affecting over 50,000 businesses nationwide. The initial phase of this plan will focus on training and upskilling more than 20,000 individuals in digital competencies. Furthermore, a substantial investment of 4.5 billion euros is projected to be injected into the economy over the next 4 years as part of these efforts (Portugal.Gov.PT, 2017).

Esmaeilian et al. (2020) suggest that by utilizing I4.0 technologies, a smart production network can achieve efficient real-time monitoring, agile communication, autonomous functionality, and smooth material flow. Technological advancements have created new opportunities for businesses to develop unique value propositions, ranging from tailored customizations to novel service innovations.

The success of businesses is now heavily dependent on digital skills, as they have become crucial for both companies and workers. This is highlighted by Digital Economy and Society Index (2022) report, which has deemed these skills as “critical” rather than “optional.” In order to adapt to the rapidly changing digital landscape, a proposal known as the “digital decade” emphasizes the importance of businesses and public services undergoing digital transformations in various aspects.

The DESI 2022 report reveals that in the year 2021, 26% of citizens in the European Union (EU) have surpassed basic digital skills and have shown proficiency in all five categories of the Digital Skills Indicator (DSI). Possessing skills beyond the basic level is essential not only for increasing competitiveness in the job market, but also for promoting the use of digital solutions in the business industry.

According to Tubis and Grzybowska (2022), I4.0 has evolved from a focus solely on factory operations to a broader digital transformation impacting both industrial and consumer markets. This shift involves implementing intelligent manufacturing processes and digitizing entire value delivery channels, extending to areas like Logistics 4.0 (L4.0). Hofmann and Rüsch (2017) argue that I4.0 offers significant opportunities for enhancing efficiency in logistics management. They emphasize that the potential of technologies like Blockchain to enable decentralized control of value chains, leading to improvements in automated production and self-optimized delivery, which reduces the need for human intervention.

Initially, research concentrated on digitizing production processes, leading to the development of the “smart factory” or digital factory concept. I4.0 technologies are now being applied to production, supply chain operations, and Maintenance 4.0. These technologies are designed to improve economic efficiency, environmental sustainability, and social contributions within logistics. Companies adopting I4.0 will digitize processes from planning to delivery, thereby enhancing logistics through improved flow management, operational optimization, and reduced lead times (El Hamdi and Abouabdellah, 2022).

### 2.2 The influence of I4.0 technology on the efficiency of logistics operations

I4.0 technology automates and streamlines logistics operations, providing tools for problem-solving and data management. The logistics sector adopts digital technologies to tackle business challenges and enhance performance. Automation and technological advancements simplify operations and offer analytical capabilities. Over the past 4 years, I4.0 has driven significant digital transformation in logistics, improving productivity and efficiency for both consumers and firms (Feng and Ye, 2021; Efthymiou and Ponis, 2021).

Digital transformation greatly improves responsiveness to demand fluctuations and enhances flexibility in supply chain and logistics operations. Information systems use advanced computing tools to efficiently process and evaluate data, analyzing business processes. I4.0 technology fosters communication and IT infrastructure growth, moving manual operations toward digitalization and reducing communication gaps. Advanced technologies optimize logistic processes, enabling companies to develop effective systems for streamlined supply chain and inventory control activities (Enrique et al., 2022; Krishnan et al., 2024).

The rapid advancement of IT and I4.0 technologies leads to digital logistics and supply chain systems, enabling personalized products and services for consumers. I4.0 technology helps logistics companies create efficient communication systems, promoting smooth interactions with management and consumers, and providing real-time supply chain updates. It also allows for automated and remote systems to control access, facilitating high-quality data collection and analysis. These automated systems enhance operational performance by improving warehouse management and delivery tracking, minimizing communication gaps, and providing quick access to relevant information (Althabatah et al., 2023).

L4.0 marks a new era in logistics management by leveraging digital advancements, transforming traditional logistics practices into modern, innovative operations. This shift presents both challenges and opportunities for organizations (Szymańska et al., 2017). A data-centric approach is essential, as the growing volume of logistics data requires investment in BI systems and the development of skills to use them effectively (Karki, 2024). BI systems enhance visibility into logistics operations, helping identify inefficiencies and enabling data-driven decision-making to improve performance. To capitalize on L4.0, organizations must invest in new technologies and adopt data-centric methodologies (Cichosz et al., 2020).

### 2.3 Logistics 4.0

**Figure 1:**
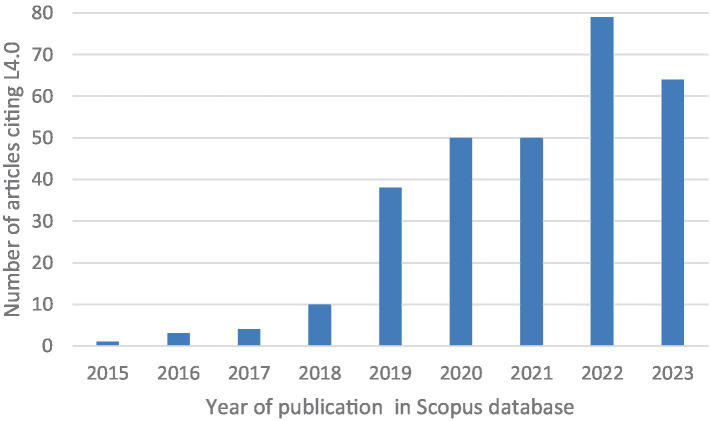
Number of articles citing L4.0 since 2015. Source Scopus database.

L4.0 involves the coordination of material and information streams within enterprises, overseeing transportation, storage, and associated data to deliver products efficiently and cost-effectively. Key logistics components include procurement, manufacturing, distribution, and reverse logistics. With rising demands for sustainability, L4.0 integrates transportation systems and provides decentralized real-time data on logistics networks. It focuses on fulfilling personalized customer needs sustainably and cost-effectively using digital technologies. Publications on L4.0 began in 2015, following the introduction of I4.0 in 2011, highlighting its role in creating responsive, customer-centric supply chains (Strandhagen et al., 2017; Winkelhaus and Grosse, 2019). Figure 1 shows the number of articles citing L4.0 since 2015 (source: Scopus database). This clearly demonstrate the growing academic interest in the topic.

Strandhagen et al. (2017) highlight the importance of L4.0 as an extension of the I4.0 concept, focusing on improvements in operational aspects such as sustainability, efficiency, and customer responsiveness. According to the same author, L4.0 impacts five key business elements: data collection and processing, assistance systems, networking and integration, decentralization and service orientation, and self-organization and autonomy.

Advanced digital technologies like Artificial Intelligence (AI), Blockchain, Cloud Computing, and BI are transforming logistics by enabling semi-autonomous operations and enhancing cross-organizational information exchange (Sigov et al., 2022). This shift affects crucial logistics functions—such as transportation, inventory management, material handling, and supply chain configuration—leading to better sustainability, traceability, efficiency, and customer responsiveness by integrating physical supply chains with digital data processes (Strandhagen et al., 2017).

L4.0, a relatively new concept, is still being explored for its full potential and benefits (Choi, 2021). Current production and logistics challenges include global market expansion, demographic changes, shorter product lifecycles, and increasing customer demands for personalized and sustainable products (Dallasega et al., 2022). Despite being new, L4.0 is gaining traction, with increasing scholarly attention since 2017 (Tubis and Grzybowska, 2022). This concept represents a major shift in supply chain management through advanced technologies and data-driven decision-making, utilizing emerging technologies like cyber-physical systems to enhance traditional logistics operations. The success of L4.0 largely depends on technological investments and capabilities (Bag et al., 2020). As logistics become more complex and data-driven, Big Data Analytics (BDA) plays a critical role in improving efficiency, flexibility, and responsiveness in logistics processes (Roshid et al., 2024).

### 2.4 Big data analytics

The distinctions between big data, BDA, and BI can be confusing. Typically, BI involves a process where raw data is collected, converted into valuable information, and used to support decision-making and drive business outcomes (Larson and Chang, 2016). With advancements in Information and communications Technology (ICT) and data storage, “raw data” has transformed into “big data.” Jin and Kim (2018) argue that BI and big data/BDA now function together as an integrated decision support system, encompassing all steps from data collection to decision-making. BDA focuses on analyzing large and varied data sets to uncover patterns, correlations, market trends, and customer preferences (Ikegwu et al., 2022). In the context of L4.0, BDA is crucial for managing the vast amounts of data generated by interconnected logistics systems and devices.

Tiwari (2020) highlights that in the era of I4.0, logistics plays a crucial role in integrating digital technologies due to its dual focus on transporting physical goods and relying on accurate, real-time data. For organizations to effectively leverage integrated cyber-physical systems, they must align their internal technology with the pace of external stakeholders like customers and suppliers, necessitating proactive investments in emerging technologies (Gupta et al., 2019; Frank et al., 2019).

Santos and Marques (2022) point out that BDA enhances managerial decision-making, especially in supply chain risk management, by enabling real-time customer needs and demand assessment. Research has shown that BDA brings several benefits to the supply chain, including better accessibility, adaptability, compatibility, connectivity, control, and coordination. Additionally, Dubey et al. (2019) found that BDA not only improves visibility in the supply chain but also builds trust among supply chain partners.

Supply chain decision-makers are focused on strategies to effectively leverage Big Data to maximize its value (Hrouga and Sbihi, 2023). Globalization, driven by just-in-time manufacturing and small batch production, accelerates industrial digitization (Sindhwani et al., 2022). Although implementing L4.0 presents challenges, the benefits—including cost savings, efficiency, and enhanced customer experiences—are significant. Zhang et al. (2021) note that few companies have successfully completed the transition to L4.0. To achieve this, decision-makers need to understand the benefits of digital technologies and their impact on logistics processes (Yang et al., 2021). BDA and BI are both crucial for contemporary data-driven decision-making.

### 2.5 Business intelligence

In today’s competitive business landscape, companies must be adaptable to address various challenges, which underscores the need for advanced decision support tools. The vast amount of available information calls for sophisticated systems to manage and utilize this data effectively.

According to Olszak and Ziemba (2007), BI systems are IT solutions designed to convert data into actionable insights and knowledge, thereby enhancing decision-making, forecasting, strategic planning, and organizational actions. Williams and Williams (2007) describe BI as a combination of methodologies and technologies that organize and analyze relevant information across business cycles to support decision-making and improve performance, including increased sales, cost reduction, and better outcomes. Agiu et al. (2014) view BI as the use of software and business applications or external data analysis to aid decision-making within a company.

BI involves two main activities: collecting information from source systems, often in its raw state, and processing it into valuable insights to support decision-making (Watson and Wixom, 2007). It includes exploring, integrating, aggregating, and performing multidimensional analysis on data from various sources (Olszak and Ziemba, 2007).

#### 2.5.1 Characteristics of BI

For BI to be effective, organizations must carefully design and implement their culture, processes, and technologies to enhance both strategic and operational decision-making (Azeem et al., 2021). BI is both a process and a product. As a process, it involves methods for generating valuable insights essential for organizational success. As a product, it provides information that helps organizations predict the behavior of competitors, suppliers, customers, technologies, and other aspects of the business environment with a reasonable level of certainty (Caseiro and Coelho, 2018).

Watson and Wixom (2007) highlight several benefits of BI, including more efficient access to high-quality data, improved analysis and decision-making, and enhanced monitoring of Key Performance Indicators (KPIs) and company strategy. BI tools also support the creation of dashboards for tracking indicators, comparing data over time, and setting objectives, which aids intuitive data analysis.

Olszak and Ziemba (2007) emphasize that BI offers significant competitive advantages over other information management systems. These include the ability to integrate diverse data, analyze large datasets, and produce comparative reports. BI also supports simulations and forecasts based on various assumptions, enabling rapid responses to market and organizational changes (Ragazou et al., 2023). The growing emphasis on BI in large companies reflects its potential to deliver substantial benefits, with BI projects increasingly associated with business information collection (Hannula and Pirttimaki, 2003).

#### 2.5.2 Benefits and barriers of BI

Zwass (1992) suggests that BI focuses on providing high-quality, current, accessible, and reliable information to meet user needs. Wright (1993) and Primak (2008) argue that effectively utilizing BI-derived information can lead to increased revenue, reduced costs, and enhanced management performance, which directly boosts organizational efficiency. According to Primak (2008), BI facilitates the preparation and processing of data, converting it into actionable information that supports organizational decision-making and delivers significant benefits to users.

According to Abukari and Jog (2003) and Primak (2008), the advantages of BI adoption include: Enhanced information reliability; Integration of data from diverse sources; Increased decision-makers’ agility; Simplified information accessibility; Enhanced competitive edge; Streamlined access control across hierarchical levels; Expedited information delivery for strategic decision-making.

A major challenge in implementing BI within organizations is cultural resistance. This resistance often arises from a reluctance to change established work practices, driven by entrenched processes and fears about job security due to technological advancements. Carvalho (2019) highlights that achieving the benefits of BI requires a strong analytical organizational structure. Abukari and Jog (2003) note that investing in BI systems presents difficulties, primarily because the benefits are often intangible, such as improvements in processes, which complicates the calculation of return on investment. Primak (2008) emphasizes the need to address these barriers for BI to succeed, stressing the importance of careful planning to avoid past failures.

Abukari and Jog (2003) and Primak (2008) also identify barriers hindering BI enhancement: Siloed departments impeding data warehouse formation; Underestimation of data and information utility; Errors in acquiring external data; Managerial unfamiliarity with BI; High costs associated with BI project implementation.

Conclusively, BI implementation is intricate, influenced by internal and external factors, with organizational responses dictating system success or failure.

#### 2.5.3 Types of BI analysis

BI analyses can be applied across various business cycles, providing valuable insights. According to Olszak and Ziemba (2007) and Williams and Williams (2007), some examples of BI-derived analyses include:

Profitability Analysis of Products and Services: By analyzing historical sales data across products, customers, and regions, along with comparing forecasted and actual sales figures, companies can assess the profitability of their products and services. This analysis helps identify sales deviations and allows businesses to optimize marketing campaigns and sales strategies for better financial outcomes.

Customer relationship analysis: Utilizing data on order history, customer lists, and satisfaction surveys facilitates analyses of customer retention levels and the performance of customer support services. Insights gained from these analyses enable actions to enhance customer retention, loyalty, and support services;

Strategic planning: Modeling various key variables pertinent to organizational development and assessing the company’s strategy, mission, and objectives;

Financial and management control: Drawing from accounting data on income, expenses, customer accounts, and budgets, analyses of budget deviations, accounts receivable aging, and income item evolution can be conducted. These analyses facilitate improvements in budgeting and forecasting processes, focusing on key deviations and identifying aged customer balances to define collection strategies.

#### 2.5.4 SWOT analysis for the industry implementing BI

A concise SWOT analysis can be provided for this study, highlighting the strengths, weaknesses, threats, and opportunities for the industry implementing the BI project. Summing up above subsections: (i) strengths include data-driven decision making, improved efficiency, competitive advantage; (ii) weaknesses encompass high implementation costs, data quality issues and resistance to change; (iii) opportunities involve growing demand for data analytics, technological advancements and expansion into new markets; (iv) threats consist of data security concerns, rapid technological changes and market competition.

The success of BI initiatives relies not just on the technology or systems themselves, but on their effective implementation, adoption, and utilization within the organization.

## 3 Methodology

### 3.1 Data and information gathering

The research initiative begins with gathering data and information on foundational concepts related to I4.0, L4.0, and BI to understand previous work and studies in these fields. The literature review focuses on understanding these concepts in depth and identifying essential technologies for digital transformation that provide a competitive edge. This exploration helps clarify the criteria companies use to invest in L4.0 technologies and shapes the research approach and identifies areas for further investigation.

The study emphasizes examining the benefits and challenges of BI implementation. While methods such as focus groups, brainstorming sessions, interviews, and surveys were considered, the Delphi method was chosen for its effectiveness in analyzing the strategic impact of BI technologies from a future-oriented perspective. The Delphi method is valuable for gaining insights into what will be relevant regarding BI technologies in this context.

To ground the research, a case study was conducted with a leading logistics operator in Portugal. Case studies are versatile and commonly used to develop, challenge, or explain theories, elucidate situations, establish solutions, and explore phenomena (Meirinhos and Osório, 2010).

### 3.2 Delphi method

The Delphi method is designed to address complex issues by organizing a structured communication framework that gathers and refines expert opinions. According to Linstone and Turoff (1975), it involves systematically collecting and synthesizing informed judgments from experts to reach a consensus over time (Williams and Webb, 1994).

The method works by distilling and combining the collective knowledge and experiences of a panel of experts through a series of questionnaires, with controlled feedback provided between rounds (Gupta and Clarke, 1996). The goal is to achieve a reliable consensus on the issue rather than just classifying or evaluating information (Dickson et al., 1984).

In summary, the Delphi method involves multiple rounds of questioning, with each round building on the previous responses to refine and converge toward a consensus. This approach allows for the aggregation of expert insights to make more accurate predictions, provides a structured way to understand and integrate expert opinions, and enables respondents to adjust their answers based on feedback from earlier rounds.

### 3.3 Delphi characteristics

The Delphi method utilizes a structured, iterative approach to gather and refine insights from experts while promoting learning among participants. It effectively collects knowledge without the challenges of face-to-face interactions, such as group conflicts or individual dominance, embodying the principle that “the whole is greater than the sum of its parts” (Gupta and Clarke, 1996).

The process involves querying a panel of experts on a specific topic through multiple rounds of questionnaires. Experts remain anonymous, and their responses are aggregated and summarized after each round. This summary is then redistributed for further feedback, with each iteration aimed at achieving a higher level of consensus (Linstone and Turoff, 1975; Williams and Webb, 1994).

Meyrick (2003) notes that the Delphi method leverages the expertise of specialists, allowing them to review and reflect on each other’s viewpoints through the iterative questionnaire process, thereby enhancing the depth and reliability of the collected insights. As for Munaretto et al. (2013), its primary characteristics, advantages, and benefits are:

Anonymity: Enhances spontaneity and openness by preventing influence from dominant or persuasive individuals, and reduces reluctance to express unpopular opinions. This helps mitigate personal biases and distortions from participant interactions, although it might lead to incomplete recall or missed viewpoints (Grisham, 2009).

Feedback: Crucial for maintaining focus on the study’s objectives, setting goals, and enabling participants to review and refine their opinions based on collective feedback. While it helps in building consensus and reducing disagreements, excessive reliance on feedback might exclude dissenting views from the analysis (Marques and Freitas, 2018; Yousuf, 2007). Feedback is a key differentiator of the Delphi method from traditional opinion polling (Linstone and Turoff, 2002).

Flexibility: Allows participants to consider peers’ opinions and arguments, which can prompt reconsideration of their own views. However, this flexibility might lead to forced consensus where participants might passively adopt others’ opinions (potentially compromising the authenticity of the consensus).

Utilization of experts: Ensures that reliable concepts and judgments are formed based on expert knowledge, though it can lead to rapid consensus formation that might not always reflect nuanced opinions.

Consensus: Aims to align expert opinions and identify reasons for disagreement. However, there is a risk of creating an artificial consensus if not managed carefully.

Interactivity: Facilitates non-hierarchical exchanges and enhances response adequacy through reciprocal learning among respondents. However, online interactions might lead to less detailed responses despite being quicker and more cost-effective, potentially limiting the depth of feedback (Grisham, 2009).

## 4 Case study

### 4.1 Company context

The case study focuses on a leading logistics operator in Portugal, a major player in the national market with over 2,500 employees and operations across the Iberian Peninsula. This large organization recently embarked on a BI project using Power BI.

Power BI, developed by Microsoft, is a robust business analytics service that offers interactive visualizations and BI capabilities. It provides a user-friendly interface for creating reports and dashboards, aiding organizations in making data-driven decisions. Key features of Power BI include data connectivity, interactive dashboards, and a range of visualization options such as charts, graphs, maps, and gauges. These tools enable the company to analyze data effectively and respond swiftly to market changes, thereby staying competitive amidst price, supplier, and customer volatility. Due to confidentiality, further details about the company’s specific operations and BI implementation cannot be disclosed.

### 4.2 Welphi plataform

In this case study, the Welphi online platform was utilized to distribute the questionnaire to respondents (Welphi, 2024). Online surveys offer significant advantages, including their non-intrusive nature, cost-effectiveness, and time savings due to the elimination of manual data entry and paper-related expenses (Wright, 2005).

However, one challenge of online surveys is the potential for low response rates. To address this, follow-up reminders can be employed, as demonstrated by Kaplowitz et al. (2004), who found that reminder emails can enhance response rates. The Welphi platform supports this approach by enabling the scheduling of reminder emails to encourage higher participation.

Before distributing the questionnaire, participants were informed through internal announcements and a comprehensive Word document detailing the study’s topic, methodology, and platform.

**Figure 2:**
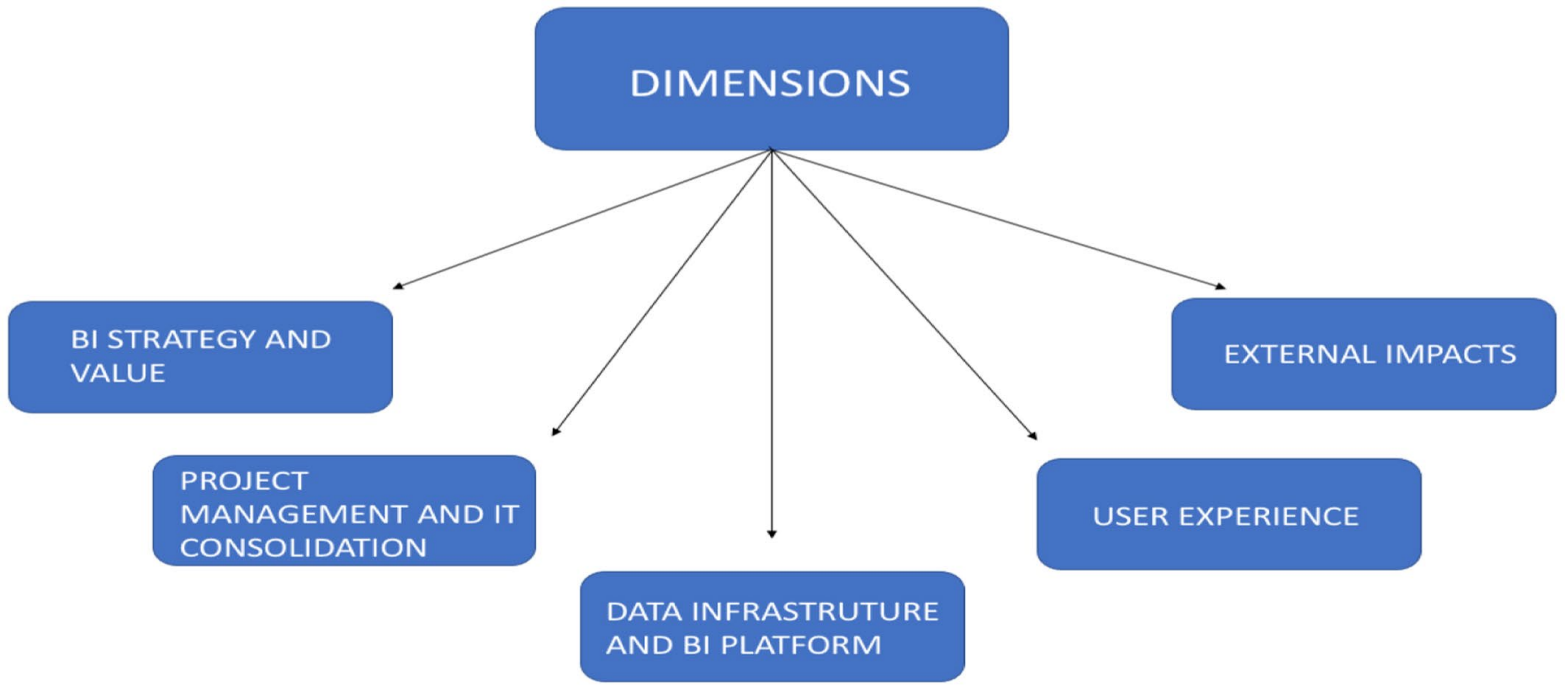
Dimensions of the questionnaire.

The Welphi platform allows for the division of questionnaires into various sections and sub-sections. The study’s dimensions scheme for the questionary are detailed in Figure 2.

The platform uses a round system to anonymously compare participants’ opinions with their peers, fostering group consensus without face-to-face interaction. Welphi supports asynchronous, online, participatory, and interactive comparisons of ideas. Anonymity encourages participants to adjust their initial positions based on group feedback, promoting consensus. Participants can respond without creating a profile or logging in.

The tool can be accessed at the user’s convenience via a link provided in the initial email, minimizing scheduling conflicts. This platform enables the creation of questionnaires to gather opinions from multiple participants who have busy schedules or are geographically dispersed, as was the case with this company. Given that the company has offices spread across Portugal, it would be challenging to bring all participants together in one location on the same day.

Welphi’s distinguishing features include the ability to create questionnaires and rounds, invite participants, set approval and rejection rules, develop response scales, share round results, and generate statistical graphs. One significant advantage is the automatic generation of statistics for result analysis. Welphi also allows the establishment of approval and rejection rules to manage consensus and decide on elements for subsequent rounds. Data can be exported to Excel for further analysis.

### 4.3 Process of implementation

The next few steps that will be described will be applied in the case study. The implementation of the Delphi method involves several steps, which, according to the consulted literature (Yousuf, 2007), can be outlined as follows: Selection of the expert panel; Development of the first questionnaire; Initial contact with experts and invitation to join the research; Distribution of the first questionnaire; Collection of responses to the first questionnaire; Qualitative analysis of responses; Preparation and distribution of the second questionnaire with feedback; Collection and analysis of responses to the second questionnaire; Comparative analysis of responses from both rounds.

#### 4.3.1 Selection of experts

Initially, the focus was on determining the number of participants, selecting experts, and structuring the questionnaire items. Due to confidentiality concerns, sociodemographic data was not collected. The study centered on five key areas within the company—logistics, transport, finance, innovation, and central services—by selecting participants from each area based on their engagement with the BI tool. This diverse sampling from various departments aimed to provide a comprehensive view and enrich the analysis of how different departments interact with the tool. The director had initially suggested involving 180 participants, but it was decided to include 61, aligning with recommendations for such studies to have between 10 and several dozen participants. According to criteria established by Skulmoski et al. (2007), selected experts should possess relevant knowledge and experience, be willing and capable to participate, have sufficient time, and demonstrate effective communication skills.

#### 4.3.2 Development of the questionary

To structure the items to be included in the questionnaire, the statements were prepared through a literature review, as well as through semi-structured meetings that took place in April 2024. To avoid the questionnaire stimulating fatigue in participants, the time available to carry out the study and the complexity of the questions, a Delphi questionnaire was created consisting of five dimensions as demonstrated in Figure 2, where within each dimension we have between three and six statements for the experts to reflect and give their opinion, in order to achieve the objectives of this research. The study used a Likert scale with five levels to gauge participants’ agreement with various statements. The scale ranged from “Strongly disagree” to “Strongly agree,” allowing participants to express their level of agreement. Additionally, participants had the opportunity to provide detailed comments or additional information on each statement. To ensure ease of understanding and response, the questionnaire was written in Portuguese, reflecting the native language of the company’s employees and avoiding potential issues with English comprehension.

The Delphi method used in this study involved two rounds of responses. The first round ran from April 29 to May 7, 2024, and the second from May 8 to May 14, 2024. The decision to limit the number of rounds to two aimed to balance the depth of feedback with the need to manage the duration and reduce the risk of participant dropout. The questionnaire was organized into dimensions relevant to the company’s interests and informed by the literature review, with Table 2 illustrating these dimensions and their sources.

**Table 2:** Dimensions and sources.

Dimension of the questionnaire	Source
BI strategy and value	Yeoh et al. (2008) and Popovic et al. (2014)
Project management and IT consolidation	Yeoh et al. (2008)
Data infrastructure and BI platform	Yeoh et al. (2008)
User experience	Hung et al. (2016)
External impacts	Premkumar and Roberts (1999)

The first dimension of the study, “BI strategy and value,” included statements designed to evaluate the perceived value and strategic alignment of the BI project. One key statement was, “The BI project will create value for the business,” which was developed from semi-structured meetings to reflect the company’s specific needs. Other statements included: “The level of BI implementation will be close to the business needs,” “Employees are aware of the critical success factors for the BI project” (El-Adaileh and Foster, 2019), “The use of this technology will be a necessity to compete in the market,” “We could lose customers to the competition if we do not adopt these new technologies” (Premkumar and Roberts, 1999), and “Managers will strongly support the adoption of these new technologies” (El-Adaileh and Foster, 2019). These statements were included based on their established relevance and discussion in the literature, ensuring they were pertinent for expert evaluation.

In the second dimension, “Project management and IT consolidation,” the focus was on understanding employee perceptions of data management and system integration. The statements included were: “BI design contributes to a more standardized data architecture” and “The set of indicators that the platform will make available will be appropriate,” based on discussions in semi-structured meetings. Additionally, the literature review supported the inclusion of statements such as “BI technology will contribute to the centralization and unification of data” and “Top management will be aware of the benefits of this new technology” (El-Adaileh and Foster, 2019).

For the third dimension, “Data infrastructure and BI platform,” the statements were aimed at evaluating the efficacy of the BI tool in meeting data needs and improving information quality. Statements like “The Power BI tool will satisfy your data integration needs” and “The BI tool will make information clearer and more accurate” were developed from semi-structured meetings. The literature review added statements such as “BI technology will improve decision-making by providing pertinent and up-to-date information” (Premkumar and Roberts, 1999; El-Adaileh and Foster, 2019) and “BI will help management optimize resources and increase productivity” (Premkumar and Roberts, 1999).

The fourth dimension, “User experience,” sought to assess user perceptions of the BI tool’s complexity and integration into existing workflows. Statements included were: “The skills required to use BI technology will be very complex,” “Integrating these technologies into our current work practices will be very difficult” (Premkumar and Roberts, 1999), and “Top management will actively encourage employees to use new technologies in their daily tasks” (El-Adaileh and Foster, 2019; Premkumar and Roberts, 1999). These statements were chosen based on previous research to ensure their relevance to the study.

In the final dimension, “External impacts,” the focus was on understanding how external stakeholders might influence the adoption and use of the BI technology. Two statements were drawn from existing literature: “Our suppliers will push for the use of this technology as a way of doing business” and “Our customers will push for the use of this technology as a way of doing business” (Premkumar and Roberts, 1999). Additionally, the statement “The data that is expected to be made available will meet the needs of customers and suppliers” was collaboratively developed by the company and the author during meetings held in April 2024.

**Figure 3:**
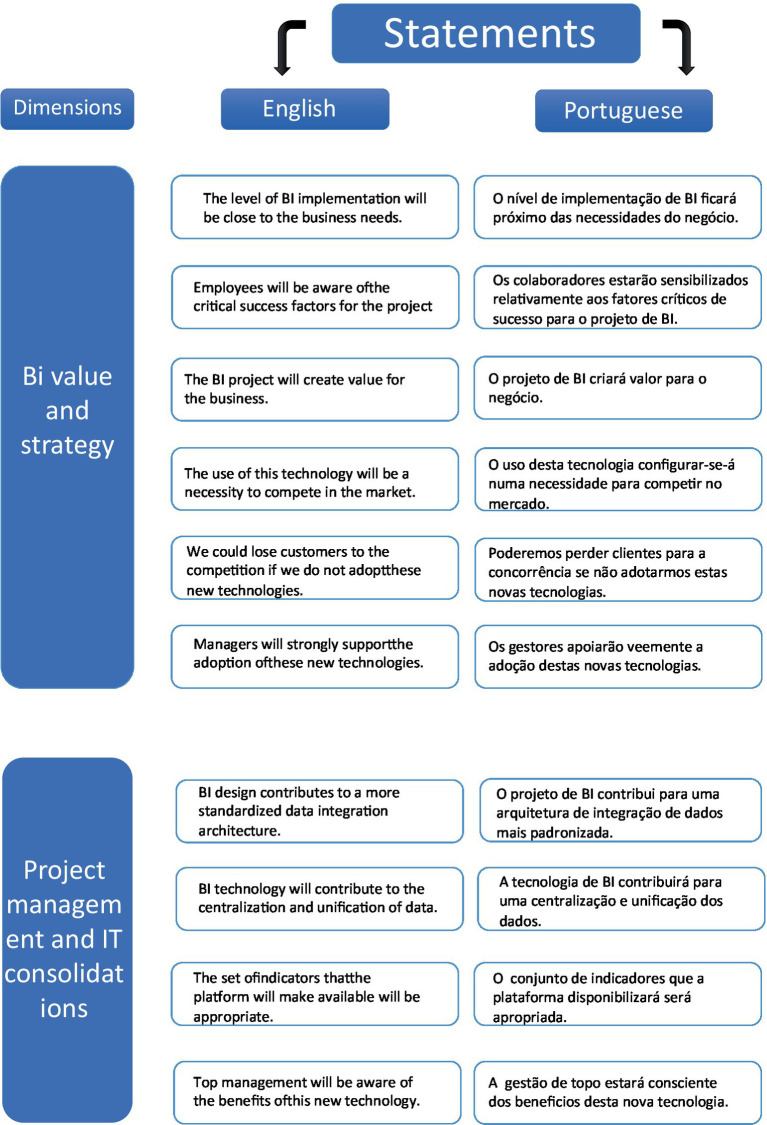
Statements in English and Portuguese from 1st and 2nd dimensions.

**Figure 4:**
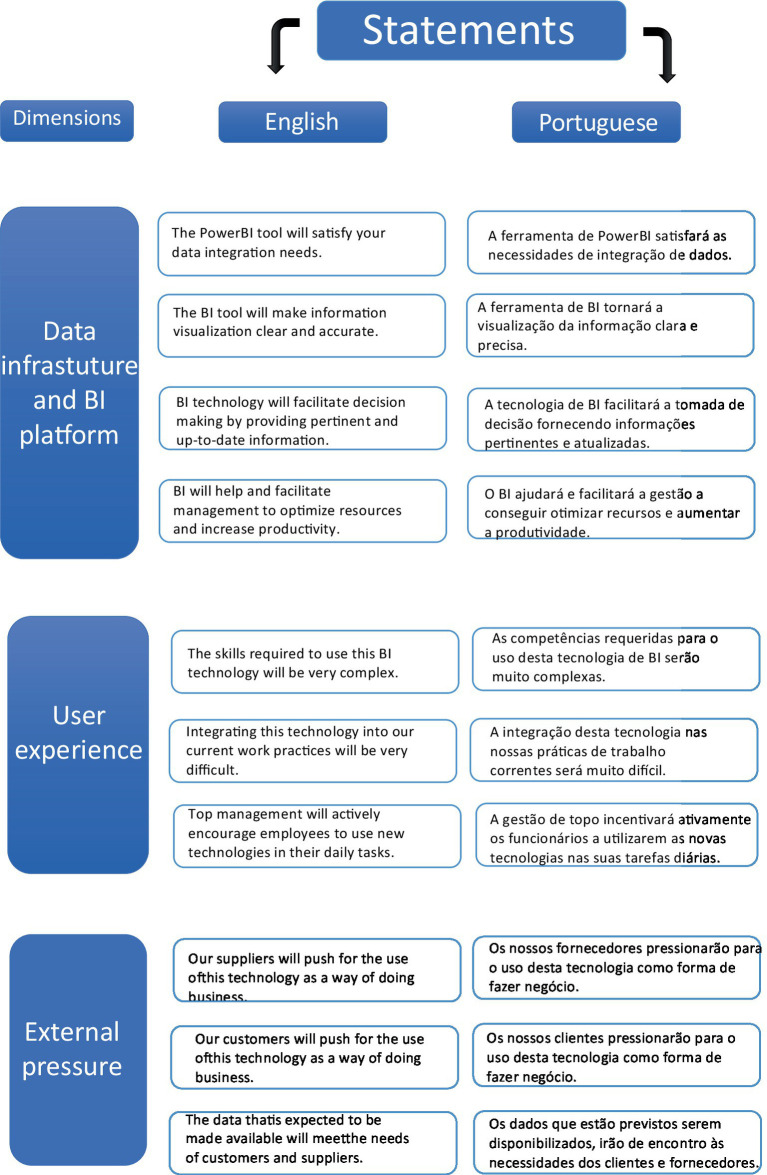
Statements in English and Portuguese from 3rd, 4th, and 5th dimensions.

Throughout this document, you will find tables extracted from Welphi that display the results from each dimension across the two rounds of the study. These tables are presented in Portuguese, but for clarity, Figure 3 will include the statements for the first and second dimensions, while Figure 4 will cover the third, fourth, and fifth dimensions. Both figures will provide the statements in both English and Portuguese to facilitate a comprehensive understanding of the findings.

#### 4.3.3 Initial contact with experts and invitation to join the research

The initial outreach to the company began with a telephone call, followed by an email to the communications department. This email detailed the intention to conduct a case study and requested contact information for someone in the innovation department.

In response, a director from the innovation department proposed a Teams call to provide a more thorough explanation of the case study process and requirements. Over the subsequent weeks, several calls and in-person meetings were conducted to finalize the details, including the selection of participants for the questionnaire.

Once all arrangements were confirmed, participants received an email invitation from Welphi to begin the questionnaire.

#### 4.3.4 Analysis and discussion of results

Data collection was carried out using the Welphi tool, which allowed experts to complete both rounds of the questionnaire at their convenience. This method avoided face-to-face contact, thereby minimizing any potential bias in their responses. The process involved 61 participants from the company selected for the case study. In the first round, 82% of the questionnaires were fully completed, with 50 out of 61 responses. In the second round, 90% of the remaining questionnaires were fully completed, with 45 out of 50 responses. Only those participants who had fully completed the first round were considered for the second round. The 50 participants included in the analysis were distributed across various departments as follows: Logistics (31 experts); Transport (10 experts); Finance (4 experts); Innovation (4 experts); Central Services (1 expert).

This cross-departmental involvement provided a comprehensive view of the business, the project, and all users. The questionnaire focused on analyzing the impact of BI on the company.

#### 4.3.5 Delphi survey: round 1

In the first round, responses from 50 out of the 61 experts were considered. Table 3 summarizes the results for each dimension, highlighting the statement with the highest percentage of agreement in this initial round. The table includes the statement, the level of agreement, and the corresponding percentage. It is noteworthy that the “Data infrastructure and BI platform” dimension featured two statements with identical agreement levels and percentages.

**Table 3:** Statements with the highest level of agreement in round one.

Dimensions	Statements	Level of agreement	Round 1
BI strategy and value	The use of this technology will be a necessity to compete in the market	Strongly agree	64%
Project management and IT consolidation	BI technology will contribute to the centralization and unification of data	Agree	66%
Data infrastructure and BI platform	The BI tool will make information visualization clear and accurate	Agree	60%
	BI will help and facilitate management to optimize resources and increase productivity	Agree	60%
User experience	Top management will actively encourage employees to use new technologies in their daily tasks	Agree	66%
External impacts	Our suppliers will push for the use of this technology as a way of doing business	Neither disagree nor agree	60%

In the subsequent subchapter titled “Delphi Survey: Round 2,” this table will be updated with an additional column to show the percentage of agreement for these statements in the second round, with the aim of assessing any increase in consensus.

While Table 3 focuses on the statements with the highest levels of agreement, it is also important to note that when aggregating responses into the “Agree” and “Strongly Agree” categories, the majority of statements achieved over 80% consensus.

As observed in Table 4, all statements received responses in the “agree” and “strongly agree” levels, with percentages ranging from 50 to 64%. The consensus reached among participants revealed that 60% agreed with the statement that the company might lose customers to competitors if it does not adopt new technologies. This reflects a broad recognition of the importance of advancing technology to meet evolving customer expectations and improve processes.

**Table 4:** Results round 1, BI value and strategy.

	Discordo totalmente	Discordo	Nem discordo nem concordo	Concordo	Concordo totalmente
O nível de implementação de BI ficará próximo das necessidades do negócio	0%	6%	12%	56%	26%
Os colaboradores estarão sensibilizados relativamente aos fatores críticos de sucesso para o projeto de BI	0%	16%	32%	50%	2%
O projeto de BI criará valor para o negócio	0%	0%	2%	42%	56%
O uso desta tecnologia configurar-se-á numa necessidade para competir no mercado	0%	0%	2%	34%	64%
Poderemos perder clientes para a concorrência se não adotarmos estas novas tecnologias	0%	4%	8%	28%	60%
Os gestores apoiarão veemente a adoção destas novas tecnologias	0%	0%	18%	58%	24%

Extracted from Welphi platform.

The expert comments provided in the survey offer additional insights into each statement within the dimensions. For instance, one statement received mixed responses, with some experts disagreeing. One expert noted a lack of internal dissemination about the BI tool, which may explain the varied responses. Another comment highlighted that many managers lack the necessary training to effectively use BI tools. This gap in training leads to slower adoption and necessitates greater efforts from the company to promote proper use. The same participant also emphasized the importance of managing managers’ expectations regarding BI tool capabilities to prevent frustrations and ensure the successful implementation of technological improvements.

In the realm of project management and IT consolidation (Table 5), it was found that there is consensus on the statements fell within the “agree” range, between 48 and 66%. Participant feedback on the BI implementation process highlighted several important considerations. One expert, who agreed with the statement that careful implementation of BI tools is essential, emphasized the need for ongoing collaboration between the project team and qualified users, as well as project managers. This collaboration should focus on continuously refining indicators and data analysis methods to align with each area’s needs, thereby maximizing the BI tool’s value to the company.

**Table 5:** Results round 1, project management and IT consolidation.

	Discordo totalmente	Discordo	Nem discordo nem concordo	Concordo	Concordo totalmente
O projeto de BI contribui para uma arquitetura de integração de dados mais padronizada	0%	0%	4%	58%	38%
A tecnologia de BI contribuirá para uma centralização e unificação dos dados	0%	0%	0%	66%	34%
O conjunto de indicadores que a plataforma disponibilizará será apropriada	0%	6%	30%	48%	16%
A gestão de topo estará consciente dos benefícios desta nova tecnologia	0%	2%	16%	50%	32%

Extracted from Welphi platform.

The expert also cautioned against potential pitfalls in the approval process, noting that approvers may lack adequate BI knowledge, which could lead to the approval of models that do not meet end users’ needs. Therefore, the project team must ensure that the BI models are suitable and effectively address user requirements.

Furthermore, the expert recommended conducting pilot tests and allowing for longer approval cycles. Emphasizing the importance of flexibility, they advised working with multidisciplinary teams to refine BI models before finalizing them. This process should involve strong communication skills, a deep understanding of the business, and sufficient time for adjustments to tailor the tool to the specific needs of the company’s departments.

In summary, the expert insights underscored that the effective use of BI tools depends on meticulous planning and collaborative implementation, ensuring that the tool meets the company’s needs and delivers value through informed decision-making.

Additionally, another expert noted that the BI tool itself does not create impact; rather, it is the decisions made based on the tool’s insights that generate value. This expert rated this perspective as “agree,” reinforcing the idea that the tool’s value is derived from its application in decision-making processes.

Concerning the third dimension, “Data Infrastructure and BI Platform,” all statements reached a consensus in the “agree” category, with percentages ranging from 50 to 60% as demonstrated in Table 6. This suggests a good chance of achieving even greater consensus in the second round.

**Table 6:** Results round 1, data infrastructure and BI platform.

	Discordo totalmente	Discordo	Nem discordo nem concordo	Concordo	Concordo totalmente
A ferramenta de PowerBI satisfará as necessidades de integração de dados	0%	8%	22%	58%	12%
A ferramenta de BI tornará a visualização da informação clara e precisa	0%	0%	8%	60%	32%
A tecnologia de BI facilitará a tomada de decisão fornecendo informações pertinentes e atualizadas	0%	4%	4%	50%	42%
O BI ajudará e facilitará a gestão a conseguir otimizar recursos e aumentar a produtividade	0%	0%	6%	60%	34%

Extracted from Welphi platform.

For the first statement, a participant agreed but emphasized the importance of staying abreast of technological advancements. The participant noted that in the future, it might be necessary to move beyond Power BI, as other applications could offer more advanced capabilities for data analysis, including predictive analytics and other tools necessary for the evolution of data usage. This highlights the fast-paced nature of technology development and the potential for new, more effective solutions than current ones like Power BI.

Another participant commented on the second statement, noting that the accuracy of information is directly linked to the quality of the data. He stresses that even with a robust BI tool, if the underlying data is flawed, the reports generated will be inaccurate. The participant marked this statement as “agree.” Another participant added that a BI tool can enhance a data-driven management culture, which will require increasingly high-quality data from core systems feeding into the BI tool. This participant strongly agreed with the statement.

Lastly, a participant agreed with the final statement and noted that BI tools allow for detailed analysis of complex supply chains and logistics networks. They can be used to monitor and improve the performance of various links in the chain, providing both micro and macro views quickly, thereby fostering improvements. However, the participant cautioned that while BI is a valuable technology, it should not be seen as a “game-changer” on its own.

As demonstrated in Table 7, consensus levels exceeding 60% were achieved, suggesting a strong likelihood of even greater agreement in the next round.

**Table 7:** Results round 1, user experience.

	Discordo totalmente	Discordo	Nem discordo nem concordo	Concordo	Concordo totalmente
As competências requeridas para o uso desta tecnologia de BI serão muito complexas	2%	62%	20%	14%	2%
A integração desta tecnologia nas nossas práticas de trabalho correntes será muito difícil	16%	62%	16%	6%	0%
A gestão de topo incentivará ativamente os funcionários a utilizarem as novas tecnologias nas suas tarefas diárias	2%	0%	14%	66%	18%

Extracted from Welphi platform.

Participants generally found that using the BI technology is not particularly complex, noting its user-friendly and intuitive nature, primarily due to its visual interface. They highlighted that while end users with minimal training can easily derive insights from the BI tool, the more challenging aspects lie in building the BI system and ensuring the accuracy of the data. Configuring and preparing dashboards, although straightforward for data retrieval, was mentioned as a more complex task, which is why some participants rated this aspect with caution.

Despite these challenges, there is a consensus that integrating the BI technology into existing work practices will be relatively straightforward. Additionally, 66% of the experts believe that effective management will play a crucial role in supporting employees to utilize the new technologies in their daily tasks.

Table 8 reveals that the statements have produced a range of responses, with consensus levels varying between 38 and 60%. The first statement, despite a broad spectrum of responses from “strongly disagree” to “agree,” shows a notable concentration of opinions at the “neither agree nor disagree” level, achieving 60% agreement. One participant who rated it as “disagree” argued that, in the short term, the tool seems more beneficial for internal use rather than for suppliers.

**Table 8:** Results round 1, external pressure.

	Discordo totalmente	Discordo	Nem discordo nem concordo	Concordo	Concordo totalmente
Os nossos fornecedores pressionarão para o uso desta tecnologia como forma de fazer negócio	2%	24%	60%	14%	0%
Os nossos clientes pressionarão para o uso desta tecnologia como forma de fazer negócio	0%	10%	32%	38%	20%
Os dados que estão previstos serem disponibilizados, irão de encontro às necessidades dos clientes e fornecedores	0%	4%	34%	58%	4%

Extracted from Welphi platform.

The second statement exhibited even less consensus, with responses spanning from “disagree” to “strongly agree” and clustering between 10 and 38%. The final statement, while also showing two minor values around 4%, indicates a stronger consensus at the “agree” level. This suggests that most participants believe the BI technology’s planned data will indeed meet the needs of both customers and suppliers in the future.

#### 4.3.6 Delphi survey: round 2

In the second and final round of the Delphi survey, only the fully completed questionnaires from the initial round were included, reducing the participant pool to 50. Despite this reduction, the dropout rate was minimal, with 45 participants providing fully answered questionnaires in the second round. This decrease in participants is typical of the Delphi method, which often leads to greater consensus in subsequent rounds. As Table 9 indicates, the second round demonstrated a higher level of agreement among responses, highlighting the effectiveness of the Delphi method in achieving consensus.

**Table 9:** Statements with the highest level of agreement in round one and round two.

Dimensions	Statement	Level of agreement	Round 1	Round 2
BI strategy and value	The use of this technology will be a necessity to compete in the market	Strongly agree	64%	73%
Project management and IT consolidation	BI technology will contribute to the centralization and unification of data	Agree	66%	69%
Data infrastructure and BI platform	The BI tool will make information visualization clear and accurate	Agree	60%	69%
	BI will help and facilitate management to optimize resources and increase productivity	Agree	60%	64%
User experience	Top management will actively encourage employees to use new technologies in their daily tasks	Agree	66%	67%
External impacts	Our suppliers will push for the use of this technology as a way of doing business	Neither disagree nor agree	60%	62%

The results emphasize the importance of the statements selected based on the literature review (as detailed in Chapter 4, Section Development of the Questionnaire). A detailed examination of each dimension will follow to identify where participant consensus aligns, which is crucial for guiding management on where to direct investments and improvements in BI technology.

Analysis of Table 9 reveals that participants who use the BI tool daily particularly value the dimensions of “Project management and IT consolidation” and “Data infrastructure and BI platform.” Consensus on these dimensions increased significantly from the first to the second round, underscoring their importance. This high level of agreement highlights the need to align project management, IT consolidation, and data infrastructure with the BI tool’s capabilities to effectively support employees.

As Table 3 indicates, the statement “The use of this technology will be a necessity to compete in the market” achieved a significant increase in consensus from the first to the second round, rising by 9% to a total of 73%. This notable shift reflects a growing recognition of the critical role of the technology in maintaining competitive advantage.

Most statements within this dimension also experienced increased levels of consensus in the second round. However, one statement, “Employees will be aware of the critical success factors for the BI project,” saw a slight decline. Specifically, consensus dropped by 3%, from 50% agreement in the first round to 47% in the second. This shift moved the response from “agree” to “neither agree nor disagree.” In the first round, 50% of participants agreed with the statement, while 32% were neutral. By the second round, agreement decreased slightly to 47%, and the neutral response increased to 36%. This decrease in consensus, though minor, is notable and suggests a shift in perception that may merit further exploration to understand the reasons behind it (Table 10).

**Table 10:** Results round 2, BI value and strategy.

	Discordo totalmente	Discordo	Nem discordo nem concordo	Concordo	Concordo totalmente
O nível de implementação de BI ficará próximo das necessidades do negócio	0%	4%	9%	64%	22%
Os colaboradores estarão sensibilizados relativamente aos fatores críticos de sucesso para o projeto de BI	0%	16%	36%	47%	2%
O projeto de BI criará valor para o negócio	0%	0%	2%	36%	62%
O uso desta tecnologia configurar-se-á numa necessidade para competir no mercado	0%	0%	2%	24%	73%
Poderemos perder clientes para a concorrência se não adotarmos estas novas tecnologias	0%	2%	4%	24%	69%
Os gestores apoiarão veemente a adoção destas novas tecnologias	0%	0%	9%	69%	22%

Extracted from Welphi platform.

In the “Project management and IT consolidation” dimension, all statements exhibited increased levels of consensus from the first to the second round. Particularly noteworthy is that the statement with the highest consensus in the first round saw further growth in the second round. Most significantly, the statement “Top management will be aware of the benefits of this new technology” experienced the largest increase in consensus, rising by 6%. This trend highlights a growing agreement on the importance of project management and IT consolidation components. The elevated consensus in this dimension underscores its critical role, reinforcing the relevance of both the statements and the overall dimension as detailed in Table 11.

**Table 11:** Results round 2, project management and IT consolidation.

	Discordo totalmente	Discordo	Nem discordo nem concordo	Concordo	Concordo totalmente
O projeto de BI contribui para uma arquitetura de integração de dados mais padronizada	0%	0%	4%	62%	33%
A tecnologia de BI contribuirá para uma centralização e unificação dos dados	0%	0%	0%	69%	31%
O conjunto de indicadores que a plataforma disponibilizará será apropriada	0%	4%	31%	53%	11%
A gestão de topo estará consciente dos benefícios desta nova tecnologia	0%	2%	11%	56%	31%

Extracted from Welphi platform.

In the “Data infrastructure and BI platform” dimension, the Delphi method resulted in greater agreement among participants’ responses, as detailed in Table 12. The second round saw a significant increase in consensus, largely influenced by feedback and group results from the first round. While all statements showed higher agreement, one statement that had already achieved high consensus in the first round saw a notable 9% increase in the second round, becoming the statement with the highest consensus. Another statement experienced a 4% increase in agreement. These results underscore the participants’ emphasis on aligning the BI tool’s capabilities with the company’s objectives, particularly in terms of data centralization and unification, to fully leverage the tool’s potential.

**Table 12:** Results round 2, data infrastructure and BI platform.

	Discordo totalmente	Discordo	Nem discordo nem concordo	Concordo	Concordo totalmente
A ferramenta de PowerBI satisfará as necessidades de integração de dados	0%	4%	24%	62%	9%
A ferramenta de BI tornará a visualização da informação clara e precisa	0%	0%	2%	69%	29%
A tecnologia de BI facilitará a tomada de decisão fornecendo informações pertinentes e atualizadas	0%	4%	2%	51%	42%
O BI ajudará e facilitará a gestão a conseguir otimizar recursos e aumentar a produtividade	0%	0%	7%	64%	29%

Extracted from Welphi platform.

In the “User experience” dimension, the Delphi method revealed an overall increase in consensus, as shown in Table 13. Participants generally perceived the integration of the new technology into their existing work practices as relatively straightforward, a view supported by comments from round one highlighting the tool’s intuitive design. Notably, the statement regarding the ease of integration experienced a significant 11% increase in consensus from the first to the second round.

**Table 13:** Results round 2, user experience.

	Discordo totalmente	Discordo	Nem discordo nem concordo	Concordo	Concordo totalmente
As competências requeridas para o uso desta tecnologia de BI serão muito complexas	2%	64%	22%	9%	2%
A integração desta tecnologia nas nossas práticas de trabalho correntes será muito difícil	7%	73%	16%	4%	0%
A gestão de topo incentivará ativamente os funcionários a utilizarem as novas tecnologias nas suas tarefas diárias	0%	0%	13%	67%	20%

Extracted from Welphi platform.

While other statements also saw increases, these were more modest. The first statement saw a 2% rise, and the last statement increased by just 1%. The limited growth of the final statement indicates a key issue: participants felt that the tool had not been adequately promoted. This suggests that top management needs to address how the tool was introduced to employees. Effective communication, clear incentives, and comprehensive training are essential to ensure that employees fully understand and can effectively utilize the technology to optimize their processes.

In the “External impacts” dimension, as detailed in Table 14, there was only a modest increase in consensus for the first two statements from one round to the next. The last statement’s consensus remained unchanged at 58%. Despite the minimal overall growth, the statement “Our customers will push for the use of this technology as a way of doing business” warrants particular attention. A comment from round one highlighted that some participants perceive the tool as more beneficial for internal company use rather than for customer-facing business transactions. While 40% of participants believe that the tool could become a key element in business dealings, it is crucial for top management to evaluate whether the technology is essential for enhancing business operations or if its primary value lies in improving customer relationships.

**Table 14:** Results round 2, external pressure.

	Discordo totalmente	Discordo	Nem discordo nem concordo	Concordo	Concordo totalmente
Os nossos fornecedores pressionarão para o uso desta tecnologia como forma de fazer negócio	0%	27%	62%	11%	0%
Os nossos clientes pressionarão para o uso desta tecnologia como forma de fazer negócio	0%	11%	29%	40%	20%
Os dados que estão previstos serem disponibilizados, irão de encontro às necessidades dos clientes e fornecedores	0%	4%	33%	58%	4%

Extracted from Welphi platform.

The Delphi rounds revealed a clear trend of increasing consensus among participants. Key insights from the study include the strategic importance of BI for maintaining competitive edge, the critical role of high-quality data in ensuring accurate BI outputs, and the necessity for strong involvement from top management in fostering technology adoption.

In the first round, responses varied significantly, which highlighted the need for further clarification and consensus-building. The iterative feedback and reflection process in the second round led to a greater alignment of opinions, demonstrating the effectiveness of the Delphi method in refining and consolidating participant views.

The findings underscore several crucial areas for the company to focus on for successful BI technology development. These include continuous improvement, which refers that he company must prioritize ongoing enhancement processes to stay abreast of technological advancements and maximize the benefits of BI technology; top management involvement, is active engagement and support from top management are vital for cultivating a culture that values data-driven decision-making and ensuring the effective adoption of new technologies; strategic alignment, as it is essential to align BI initiatives with the company’s strategic goals to ensure that the technology supports business objectives and enhances competitive advantage.

In summary, the study highlights that for BI technology to be effective, it must be continuously improved, supported by top management, and aligned with the company’s strategic direction.

## 5 Discussion

The results highlighted the essential role of BI technologies in enhancing the company’s competitive edge through improved decision-making aligned with its goals and objectives. Support from top management and external competitive pressure were identified as crucial factors. Top management’s vision and commitment to innovation are vital for securing the necessary resources and support for implementation, consistent with findings from previous studies (Premkumar and Roberts, 1999).

Based on the results, effective adoption of BI technologies requires aligning BI initiatives with the company’s strategic goals to ensure that the technology supports business objectives and enhances competitive advantage. IT infrastructure ensures that users receive information and data with appropriate levels of reliability, timeliness, accuracy, confidentiality, and security. It also supports the ability to adapt processes to evolving business needs and directions (Primak, 2008).

The findings emphasize several key areas for the company to focus on for successful BI technology development. One of these is continuous improvement, meaning the company must prioritize ongoing enhancement processes to keep up with technological advancements and fully leverage the benefits of BI technology. This aligns with prior studies that have identified continuous improvement as a significant factor for initiating many innovations. Firms adopt BI technology only if they perceive a need to overcome a performance gap or exploit a business opportunity (Premkumar and Roberts, 1999).

## 6 Conclusion

### 6.1 Conclusion of the study

The study aimed to assess how BI technologies are integrated into the company’s strategy and their impact on competitive advantage. The results revealed that employees view BI as essential for enhancing the company’s competitive edge through improved decision-making and strategic planning, aligned with its goals and objectives, based on accurate data. Future strategies will need to focus on developing BI technologies to continue creating business value.

Participants emphasized the importance of aligning BI tools with company objectives by centralizing and unifying data, which is critical for gaining insights that support strategic decisions. This alignment suggests that top management should prioritize improvements in project management and IT consolidation.

A key challenge identified was the lack of employee awareness regarding the critical success factors of the BI project. Feedback from the second round of the study highlighted insufficient internal communication about the BI tool, indicating an area for management to address. The study also underscored the necessity of BI indicators and the need for a continuous improvement process to keep up with technological advances and fully utilize BI capabilities.

A potential statement for the questionnaire could be: “Company provide incentives, such as courses or training, for my development in BI” This is in light of the observation that the company’s communication regarding BI technology was found to be inadequate.

In summary, the study highlights the significant benefits of BI technologies in strategic implementation and development. It stresses the transformative potential of BI in the logistics industry and emphasizes that successful implementation requires meticulous planning, ongoing collaboration, and a commitment to technological advancement.

### 6.2 Limitations of the study

This study has several limitations that should be acknowledged. As this is a case study, caution should be exercised when generalizing the findings and conclusions of this study, and the confidentiality of sociodemographic data makes it difficult to characterize the company and its employees. Firstly, given more time, it would have been beneficial to conduct the questionnaires in bilanguage (English and Portuguese) since this study is all written in English. Secondly, the online data collection method sometimes resulted in a low response rate, requiring follow-up emails to prevent high absenteeism.

### 6.3 Future research directions

Future research could include a more diverse group of experts, especially from the company’s offices in Spain. Comparative studies across different organizations in the transport and logistics sector could provide a broader understanding of the implementation and impact of BI technology. Longitudinal studies tracking BI technology adoption over time would capture technological advancements and market changes, offering insights into the long-term benefits and challenges. Employing mixed methods, such as combining quantitative surveys with qualitative interviews, would provide a holistic view of BI technology’s impact and user experiences.

## Data Availability

The raw data supporting the conclusions of this article will be made available by the authors, without undue reservation.
